# Minimal Required Resolution to Capture the 3D Shape of the Human Back—A Practical Approach

**DOI:** 10.3390/s23187808

**Published:** 2023-09-11

**Authors:** Mirko Kaiser, Tobia Brusa, Marco Wyss, Saša Ćuković, Martin Bertsch, William R. Taylor, Volker M. Koch

**Affiliations:** 1 Biomedical Engineering Lab, Bern University of Applied Sciences, 2501 Biel, Switzerland; 2 Laboratory for Movement Biomechanics, ETH Zurich, 8092 Zürich, Switzerland

**Keywords:** three-dimensional imaging, back surface, trunk asymmetry, back shape analysis, depth camera, trunk surface reconstruction

## Abstract

Adolescent idiopathic scoliosis (AIS) is a prevalent musculoskeletal disorder that causes abnormal spinal deformities. The early screening of children and adolescents is crucial to identify and prevent the further progression of AIS. In clinical examinations, scoliometers are often used to noninvasively estimate the primary Cobb angle, and optical 3D scanning systems have also emerged as alternative noninvasive approaches for this purpose. The recent advances in low-cost 3D scanners have led to their use in several studies to estimate the primary Cobb angle or even internal spinal alignment. However, none of these studies demonstrate whether such a low-cost scanner satisfies the minimal requirements for capturing the relevant deformities of the human back. To practically quantify the minimal required spatial resolution and camera resolution to capture the geometry and shape of the deformities of the human back, we used multiple 3D scanning methodologies and systems. The results from an evaluation of 30 captures of AIS patients and 76 captures of healthy subjects showed that the minimal required spatial resolution is between 2 mm and 5 mm, depending on the chosen error tolerance. Therefore, a minimal camera resolution of 640 × 480 pixels is recommended for use in future studies.

## 1. Introduction

Adolescent idiopathic scoliosis (AIS) is an abnormal spinal deformity in adolescents. Scoliosis is defined as a lateral curvature of the spine with a Cobb angle greater than 10 degrees. The Cobb angle is the angle of the lateral curvature between the two most tilted vertebrae. If AIS is left untreated, it can lead to cosmetic changes, pulmonary dysfunction, and pain [1]. To detect AIS, some countries have introduced school screening programs where visual assessments, scoliometers, or 3D scanners are used to estimate the primary Cobb angle [2]. In these screening programs, adolescents are evaluated to determine whether a full radiograph is necessary for further examination. Depending on the severity or risk of progression, AIS patients undergo a radiograph every 6 months [3]. Modern X-ray imaging systems, especially the EOS imaging system, significantly reduce ionizing radiation [4]. However, such systems are very expensive, and several studies show that cancer incidence in AIS patients is five times higher than that in healthy adolescents [5]. As an alternative approach, rasterstereography is investigated and implemented worldwide, but there remains a gap between the estimations using rasterstereography and results obtained using radiographic imaging [6]. With the advances in modern 3D imaging systems, this topic has experienced a recent revival, especially for low-cost consumer 3D capturing systems such as Kinect from Microsoft, Xtion Pro from ASUS, Intel D415 from Intel, Orbbec Astra from Orbbec, and many more. Kinect was proposed and used to obtain the spinous process line and spine midline [7,8,9]. The methods that the authors used are based on a back shape curvature analysis, i.e., Gaussian curvature and mean curvature, and the anatomical landmarks used to estimate the spine midline are characterized by specific trunk surface curvature. Xtion Pro was used to obtain an asymmetry map and index via Adam’s bending test, which is often performed in visual assessments of AIS patients [10,11]. The asymmetry index is based on the root mean square error (RMSE) between the captured 3D back shape and a mirrored version. The authors demonstrated a high correlation between the asymmetry index and the Cobb angle. The Cobb angles obtained using DIERS formetric 4D (DIERS International GmbH, Schlangenbad, Germany) were compared with those obtained using radiography [12]. The DIERS formetric 4D uses rasterstereophotogrammetry to reconstruct the shape of the back. Curvature analysis, i.e., a curvature map, is used to detect various anatomical landmarks and symmetry lines. This symmetry line divides the back into two halves with minimal left–right asymmetry and estimates the spinous processes line used to estimate 3D spinal alignment [13]. Mobile phone RGBD capturing, i.e., iPhone 13’s LIDAR, in combination with the use of a Spine Priors Model and deep learning, has been proposed [14]. The proposed method fits the Spine Priors Model to the spine-related features on the back surface. The features used include the back surface spinous curve and the back surface local symmetry close to the spinous curve. The spinous curve is automatically detected using deep learning (HRNet) and nearby local symmetry as mirror points on the lines perpendicular to the spinous curve. Thus far, no research has explored the minimal required spatial resolution and the corresponding camera resolution to capture the geometry and shape of the human back. The minimal required spatial resolution defines how many sample points are required on the human back to capture all relevant features. The minimal required camera resolution is the corresponding camera resolution in pixels necessary to satisfy the minimal required spatial resolution.

**Figure 1:**
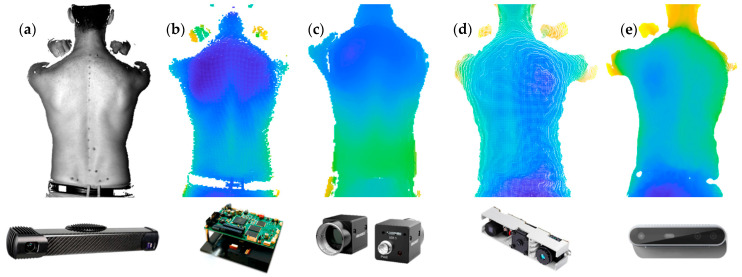
Three-dimensional back shape captures from (**a**) Photoneo MotionCam-3D; (**b**) structured light with TIDA-00254; (**c**) active stereo with BoofCV; (**d**) Orbbec Astra Mini; (**e**) Intel D415.

In this paper, we address this issue and present a practical approach for identifying the minimal resolution required to capture the topography of the human back. We used multiple 3D scanning methodologies and systems to acquire a quantification of the minimal resolution required to capture the deformities of the human back (Figure 1). In this paper, we also show that using systems that do not meet the minimal required resolution will lead to suboptimal results.

## 2. Materials and Methods

Our evaluation is based on a dataset [6] of scoliotic patients (AIS) obtained from the University Children’s Hospital Basel (UKBB, Basel, Switzerland), which consists of 30 optical captures of the human back. Data collection was approved by the cantonal Ethics Committee (EKNZ 2020-02496). The mean age of the AIS patients was 13 ± 3 years. The capturing was carried out using an Artec Eva 3D optical scanner (Artec 3D, Luxembourg). A second dataset containing 76 healthy subjects (adults without any diagnosis or surgical treatments of a spine pathology) was acquired, and this was approved by the ETH Zurich Ethics Commission (EK 2022-N-179). The mean age of the healthy subjects was 54 ± 16 years. The capturing was carried out using different 3D acquisition systems (Figure 1): Photoneo MotionCam-3D M+ [15], structured light (SL) with TIDA-00254 (DLP Lightcrafter 4500) [16] and a monochrome 2D camera from HIKROBOT (MV-CA023-10UM) [17], active stereo (AS) with Lightcrafter 4500 and two monochrome 2D cameras from HIKROBOT, single-shot structured light with the Orbbec Astra Mini [18], and active stereo with Intel D415 [19]. The Photoneo MotionCam-3D is an expensive industrial 3D scanner that produces very accurate 3D representations (3D point clouds), and thus is used as a reference and ground truth. TIDA-00254 is a DLP light engine and is used in combination with structured light (machine vision application from Texas Instruments) and active stereo (BoofCV). Intel D415 and Orbbec Astra Mini are two of the latest low-cost consumer systems. Data were processed in MATLAB R2022a on an HP Elitebook 840 G8 (Intel i7-1165G7, 16GB RAM). We provide the full code with a sample for illustration purposes (see Supplementary Materials).

Data were processed using three approaches. The first approach (Approach A) uses a frequency analysis of a ground-truth 3D representation to obtain an initial estimate of its contained frequencies. This estimate was used in the second and third approaches as the initial cut-off frequency. The second approach (Approach B) compares the shape of the reduced-quality 3D representation with the ground-truth shape, and the third approach (Approach C) compares the symmetry line of the reduced-quality 3D representation with its benchmark.

Approach A—Frequency analysis of the ground-truth 3D representation

**Figure 2:**
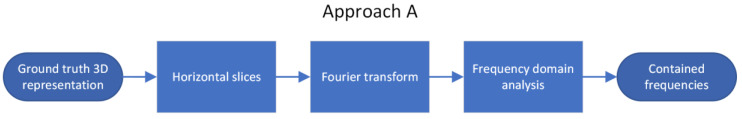
Approach A—Frequency analysis of ground-truth 3D representation.

**Figure 3:**
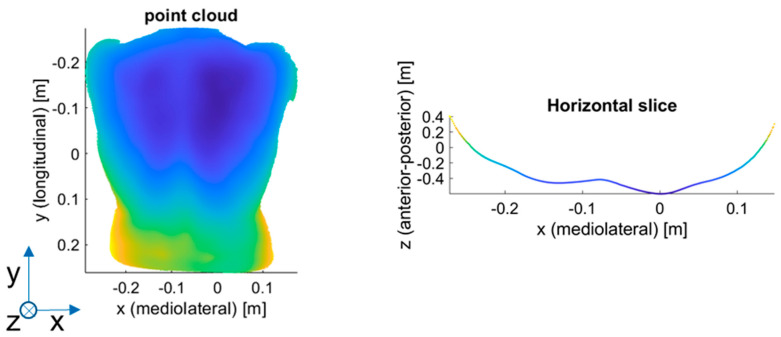
Process to calculate the horizontal slices from the ground-truth 3D representation: (**left**) ground-truth 3D representation; (**right**) horizontal slice.

The first approach is generic and can be applied to many research questions concerning the shape analysis of specific objects of interest. Approach A involves the Fourier transform of the object of interest into the frequency domain, where its contained frequencies can be analyzed. In our case, the frequency analysis process is as follows (Figure 2): the ground-truth 3D representation is sliced into multiple horizontal slices (Figure 3). Each slice is then represented as a function z=f(x), where z is the depth value, and x is the horizontal axis from left to right. The spacing between the sampling points in the x-direction is regularized. This is necessary for the Fourier transform. The fast Fourier transform (fft function in MATLAB) can then be directly applied to the function z=f(x). The Fourier transform provides the frequency domain representation as the output.

According to the Nyquist theorem, a sampling frequency (in our case, a spatial sampling frequency in [1/mm]) of twice the maximal frequency contained in the signal must be used [20]. The object of interest in our case is the human back, which is mostly a smooth surface, and thus its contained spatial frequencies are limited. The Nyquist theorem only holds true for a perfect signal without noise, measurement error, influences from the camera systems, etc. In practice, every camera system introduces additional frequencies through sampling, artifacts, and noise, especially in the higher frequency range. To keep this influence to a minimum, the camera system must be of high quality in order to produce a ground-truth 3D representation. Furthermore, these additional frequencies have to be considered when interpreting the resulting frequency-domain representation by checking the magnitudes of all frequencies. The lower frequencies with the highest magnitudes correspond to the captured back shape. Approaches B and C heavily rely on low-pass filtering, and thus the high-quality camera system can be modelled as a black box. For practical purposes, its influence on higher frequencies in the signal can be neglected. The results from approach A provide a good basis for further evaluation with approaches B and C.

Approach B—Comparison of the shape of reduced-quality 3D representation with the ground-truth shape

**Figure 4:**
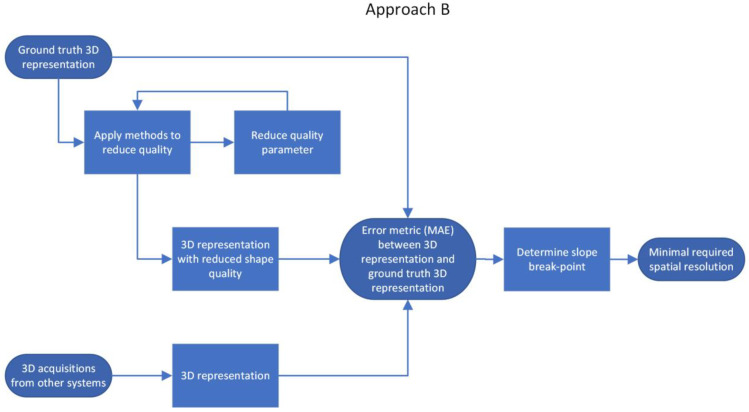
Approach B—Shape quality reduction to calculate the minimal required spatial resolution.

**Figure 5:**
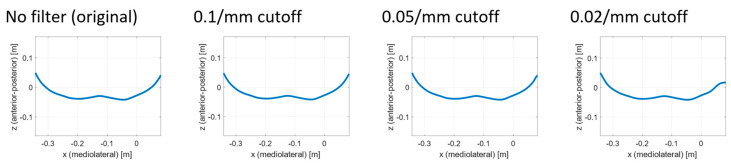
Example of shape quality reduction for a horizontal slice using a Butterworth low-pass filter.

The second approach is also generic and can be applied to other research scenarios with respect to the minimal required resolution of optical 3D acquisition systems. Approach B takes a ground-truth 3D representation of the object of interest (in our case, the human back) as the input. The ground-truth 3D representation can be either captured using a very accurate 3D acquisition system (in our case, Photoneo MotionCam-3D, with a point size of 0.52 mm, @z = 900 mm, and accuracy < 0.3 mm) or delivered as a CAD representation (e.g., for artificial objects). An evaluation metric is then calculated using the ground-truth 3D representations to obtain the baseline (Step B1). The chosen evaluation metric is the mean absolute error (MAE) between the 3D representation and baseline 3D representation. The next step (Step B2) is to apply different methods to artificially reduce the quality of the 3D representation. We used downsampling (pcdownsample method in MATLAB), reduced the spatial frequency with a low-pass Butterworth filter (butter and filtfilt methods in MATLAB), limited the depth resolution (*z*-axis), and added both random spatial noise and sinusoidal spatial noise. The full list of all methods used to artificially reduce quality is presented in Table 1. We chose the reduction in spatial frequency as our main parameter because, according to the Nyquist theorem, there is a direct and strong relationship between the minimal required resolution and the frequencies to be captured. Furthermore, the reduction in spatial frequencies showed the strongest influence on the chosen error metric. The shape quality reduction process is as follows (Figure 4): the highest frequency from approach A is used as the starting cut-off frequency for the Butterworth low-pass filter [21]. With each chosen cut-off frequency, all 3D representations are filtered with the low-pass filter (Figure 5), and the MAE between each filtered 3D representation and its corresponding ground-truth 3D representation is used as the error metric. Next, the cut-off frequency is reduced, and the procedure is repeated (Step B3). The level of reduction in frequency depends on the starting frequency, the level of desired accuracy of the final result, and the available resources for simulation. The result is a function of MAE values depending on the cut-off frequency. Reducing the cut-off frequency leads to a slow but steady increase in MAE values. As soon as the cut-off frequency reaches lower frequencies, which are essential for the 3D representation, the error metric starts to increase rapidly. In the context of this paper, this is called the slope break-point and is determined using the error tolerance chosen by the user.

The last step (Step B4) is to calculate the MAE from all 3D representations captured using all other camera systems compared to the corresponding ground-truth 3D representation. This was carried out to practically evaluate our artificial findings using existing 3D acquisition systems. The error value can then be compared to the simulated approach and classified accordingly.

Approach C—Comparison of the symmetry line of the reduced-quality 3D representation with its benchmark

**Figure 6:**
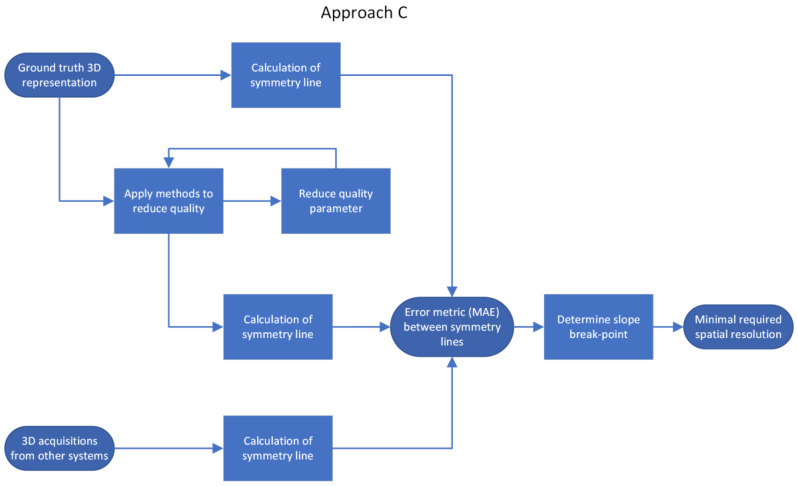
Approach C—Differences in symmetry lines calculated via reductions in shape quality.

**Figure 7:**
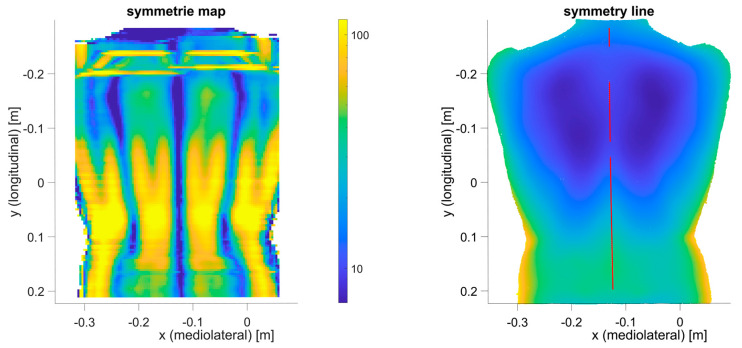
Process for calculating the symmetry line from the ground-truth 3D representation: (**left**) symmetry map, e.g., evaluation of symmetry function for each horizontal slice (Figure 3); (**right**) resulting symmetry line (red).

**Figure 8:**
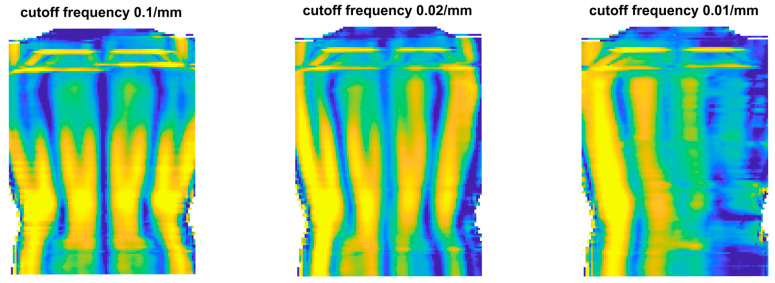
The influence of shape quality reduction on the symmetry map (symmetry line).

The third approach is application-specific, which has to be selected according to a specific research question. Our research project aims to estimate the internal spinal alignment (i.e., the line that passes through the centroids of vertebral bodies) from the human back shape (from back shape to spine approach). In the literature, there are two main approaches to address this problem. The first is a so-called asymmetry map [10], where the left side of the human back is compared to the right side of the back. The differences between the left and right sides lead to an asymmetrical map. This approach requires the back shape of sufficient quality (minimal required spatial resolution) and thus is already covered by approach B. The second is the so-called symmetry line [22]. This is very similar to the asymmetry map, where the left and right sides are compared with each other, except here we are only interested in the mid-line, the line with maximal symmetry. Ideally, this line coincides with the line above the spinous processes of the spine. Based on this symmetry line, the internal spinal alignment is estimated [23,24,25,26,27,28,29]. Since this approach does not require the full 3D shape of the human back in full detail, the procedure is as follows (Figure 6): First, the symmetry line is calculated for the ground-truth 3D representation to obtain a benchmark symmetry line (Step C1). We chose the symmetry line algorithm according to Drerup/Hierholzer [29,30] due to its simplicity. Most modern symmetry line algorithms are based on similar curvature analysis of the back shape. To calculate the symmetry line, the 3D representation is sliced into multiple horizontal slices. Each horizontal slice is then written as a function, z=f(x), where z is the depth value and x is the horizontal axis. For each horizontal slice, the symmetry point is calculated according to the formula presented in [30]. The vertical connection of each symmetry point from each slice results in the symmetry line (Figure 7). Second, the different methods from approach B are applied to artificially reduce the quality of the 3D representation (Figure 8) and calculate the symmetry line for each representation once more (Step C2). The MAE of the difference between the symmetry line from the reduced-quality 3D representation and the corresponding ground-truth 3D representation is used as the error metric. The last step (Step C3) is to repeat the procedure with the 3D acquisitions from the different camera systems.

Practical oversampling

According to the Nyquist theorem, a sampling frequency of twice the maximal frequency contained in the signal must be used [20]. The Nyquist theorem only holds true for a perfect signal, and thus, in practice, an oversampling with respect to the minimal required resolution is required. In the literature, an oversampling of at least five times the required sampling frequency is recommended for similar practical applications [31,32,33,34,35]. This level of oversampling leads to a required sampling frequency ten times higher than the highest frequency of interest.

Minimal spatial resolution

Approaches A, B, and C deliver a function $e=f(f_{c})$, where for approach A, e is the frequency magnitude and $f_{c}$ is the frequency, and for approaches B and C, e is the error and $f_{c}$ is the cut-off frequency of the low-pass filter. These functions exhibit an exponential behavior, whereas, for higher cut-off frequencies, the error increases steadily but slowly, and for lower cut-off frequencies, the error grows exponentially. The definition of the minimal required spatial resolution depends on the acceptable error, i.e., the cut-off frequency where the error is still within an acceptable range; thus, this is a somewhat practical design choice.

Minimal camera resolution

Approaches B and C can be used to define the minimal required spatial resolution. The last step is to translate this result into the minimal required resolution for the 3D capturing system. The equations for the minimal required resolution of the 3D system are as follows:

(1) $$r_{c,min,x}=\frac{W_{ROI}}{\left(c_{f}\times R_{s,min,x}\right)}$$

(2) $$r_{c,min,y}=\frac{H_{ROI}}{\left(c_{f}\times R_{s,min,y}\right)}$$

where $r_{c,min}$ is the minimal required resolution for the 3D system in pixels, $W_{ROI}$ and $H_{ROI}$ are the width and height of the region of interest (in our case, the human back), $c_{f}$ is the fill factor (ratio between the image region covering the object of interest and background), and $R_{s,min}$ is the minimal required spatial resolution. The width and height of the region of interest is found in literature, especially NASA published various parameters regarding anthropometry [36], including the width and height of the human back. The fill factor depends on the field of view (FOV) of the camera and the distance between the 3D capturing system and the object of interest. Ideally, the fill factor is close to 1 to optimize the resolution of the 3D capturing system.

## 3. Results

**Approach A—Frequency analysis of the ground-truth 3D representation**

**Figure 9:**
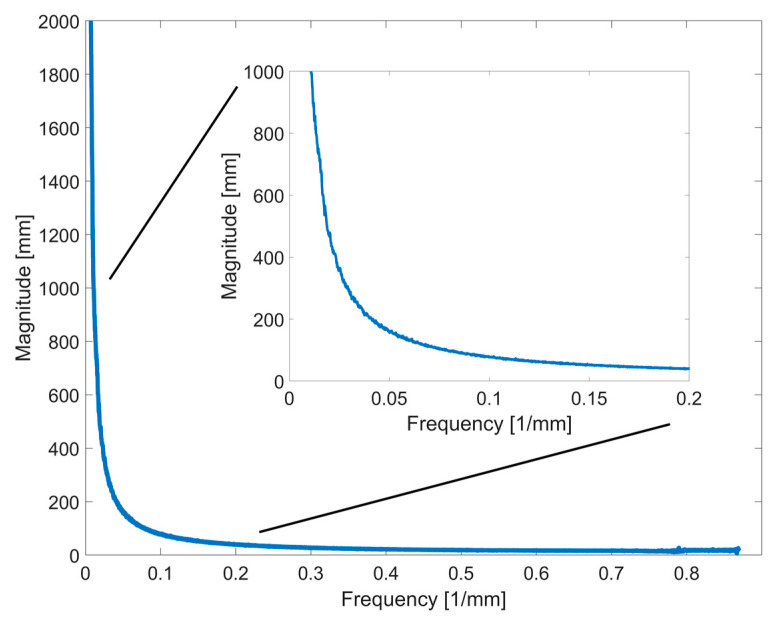
Magnitude of frequencies from the human back with zoom in on the lower frequencies.

The resulting function from the Fourier transform of 30 AIS and 76 healthy optically digitalized human backs is shown in Figure 9. The ground truths were captured using Artec EVA 3D (AIS patients) and Photoneo MotionCam-3D (healthy subjects). The highest contained frequencies are around 0.87 mm^−1^, corresponding to the capturing limit of the Photoneo MotionCam-3D. The most power (magnitude of frequencies) is contained in frequencies smaller than 0.1 mm^−1^, resulting in a wavelength of 10 mm. According to the Nyquist criteria, in theory, a sampling frequency of 0.2 mm^−1^ is sufficient to capture the relevant frequencies contained in the signal. This translates to one sampling point every 5 mm. The Nyquist criteria, however, is only valid for a perfect signal without noise, and thus, in practice, oversampling must be used.

Some studies propose a practical oversampling of five times (×5) for similar applications [31,32,33,34,35]. With this, the required sampling frequency is 1 mm^−1^, which translates to one sampling point every millimeter (oversampling of at least five times leads to a frequency that is ten times higher). The resulting frequency of 0.1 mm^−1^ is used as the initial cut-off frequency for approaches B and C.

**Approach B—Comparison of the shape of the reduced-quality 3D representation with the ground-truth shape**

**Figure 10:**
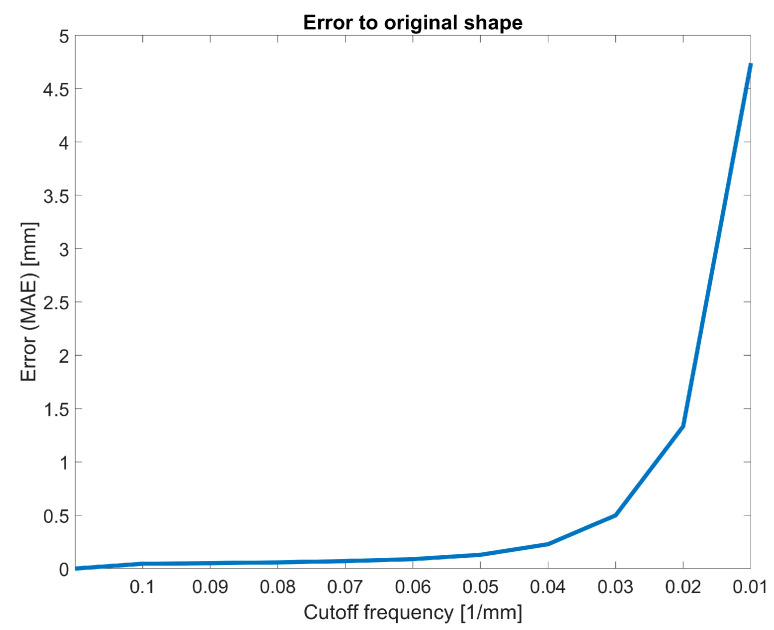
Error from the shape of a reduced-quality 3D representation compared to the ground-truth shape.

The resulting function of the shape quality reduction process is shown in Figure 10. At a cut-off frequency of 0.1 mm^−1^ from approach A, the error (MAE) is small (0.04 mm). With a cut-off frequency of 0.05 mm^−1^, the error remains small (0.13 mm). Applying a five-times oversampling to this cut-off frequency results in a required sampling point every 2 mm. With a cut-off frequency of 0.02 mm^−1^, the error starts to increase exponentially (1.33 mm). Applying a five-fold oversampling to this cut-off frequency results in a required sampling point every 5 mm. Therefore, the slope breakpoint is between 0.05 mm^−1^ and 0.02 mm^−1^.

**Approach C—Comparison of the symmetry line of the reduced-quality 3D representation with its benchmark**

**Figure 11:**
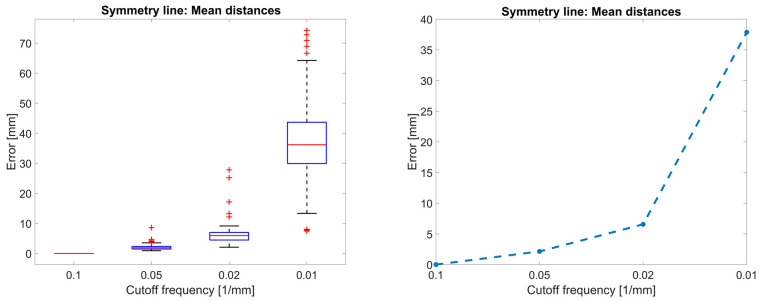
Error from symmetry line of reduced-quality 3D representation compared to its benchmark symmetry line.

The resulting function from the comparison of back symmetry lines from reduced-quality 3D representations and the ground-truth representation is shown in Figure 11. The symmetry line algorithm is inherently sensitive to high-frequency noise because it is based on the curvature analysis of a shape, and thus approach C is only evaluated with a cut-off frequency of 0.1 mm^−1^ and lower. The largest cut-off frequency of 0.1 mm^−1^ was used as a reference, and thus the error (MAE between symmetry lines) is 0 mm. With a cut-off frequency of 0.05 mm^−1^, the error is small (2.1 mm). Applying a five-times oversampling to this cut-off frequency requires a sampling point every 2 mm. With a cut-off frequency of 0.02 mm^−1^, the error starts to increase exponentially (6.6 mm). Applying a five-fold oversampling to this cut-off frequency requires a sampling point every 5 mm. Therefore, the slope breakpoint is between 0.05 mm^−1^ and 0.02 mm^−1^.

**Minimal spatial resolution**

Approach A results in the minimal required spatial resolution to capture all relevant frequencies of the human back at one sampling point every 1 mm. Since this is only an approximation from the lower frequencies with the highest magnitudes, and because the influence of the camera system is modeled as a black box, this result is only used as an initial estimation for approaches B and C. Approaches B and C result in a minimal required spatial resolution of one sampling point every 2 mm to 5 mm.

**Minimal camera resolution**

NASA states the 95th percentile of the waist back of an American male as 51.6 cm and of the interscye as 45.4 cm, and the 95th percentile of the waist back of a Japanese female as 41.0 cm and of the interscye as 39.0 cm [36]. Equation (1) results in

$$r_{c,min,x}=454\;mm/\left(0.67\times \left(2\;mm-5\;mm\right)\right)=136\;px-339\;px$$

for the horizontal resolution, and Equation (2) results in

$$r_{c,min,y}=516\;mm\;/\;\left(0.67\times \left(2\;mm-5\;mm\right)\right)=154\;px-385\;px$$

for the vertical resolution. The fill factor of 2/3 (0.67) was determined using practical measurements. The measurements for the American male are taken as the limit because the width and height of the male back are larger.

## 4. Discussion

The optical 3D systems evaluated in this paper are summarized in Table 2.

All the systems satisfy the required minimal resolution to capture the human back. The Photoneo MotionCam-3D also satisfies the minimal required spatial resolution. The results confirm that the Photoneo MotionCam-3D is a valid choice as a ground-truth-capturing device because the accuracy error of less than 0.3 mm is three times higher than the required spatial resolution resulting from approach A (1 mm) and seven to seventeen times higher than the required spatial resolution resulting from approaches B and C (2 mm to 5 mm). Texas Instruments does not specify the accuracy of the structured light and active stereo system with TIDA-00254 and HIKROBOT cameras. Practical tests conducted by the authors revealed error values of around 1 mm. Orbbec Astra Mini has an accuracy error of less than 3 mm at a 1 m distance. The required spatial resolution resulting from approach A is not satisfied; however, depending on the design choice for approaches B and C, Astra Mini satisfies the minimal spatial resolution required to capture the human back. Intel D415 does not satisfy the minimal spatial resolution required to capture the human back. An error of 2% at a distance of 1 m results in an error of up to 20 mm.

The presented results are based on the assumption of five-times practical oversampling. Depending on the design choice for the allowed error for approaches B and C, and the relaxation of practical oversampling (Table 3), both low-cost consumer systems can satisfy the minimal required resolution. For Orbbec Astra Mini, this is already the case when considering a practical oversampling of three times. For Intel D415, this is only the case if we use the theoretical Nyquist criteria and allow for the upper bound on the minimal required spatial frequency resulting from approaches B and C (0.02 mm^−1^). Depending on the acceptable error, both Orbbec Astra Mini and Intel D415 can be used to capture the human back, but the authors do not recommend using Intel D415. Astra Mini is also only recommended if the low price of the 3D capturing system is weighted higher than the quality of the results. The Photoneo MotionCam-3D and SL/AS with THE TIDA-00254 projector and HIKROBOT cameras are all valid options for capturing the human back.

The fill factor (the ratio between the image region occupied by the object of interest and the background) of 0.67 was determined using practical measurements. According to NASA, the ratios between the 95th percentile of the waist back and interscye are 1.05 (Japanese female) and 1.14 (American male). The aspect ratio of the systems under evaluation ranges from 1.33 to 1.78, resulting in a maximal possible fill factor of 0.75. However, the human back is not rectangular, and thus the fill factor is lower in practice. To better optimize the field of view of the camera in terms of pixel occupancy, a camera with an aspect ratio closer to 1 is required. Off-the-shelf systems tend to have aspect ratios of 1.33 or higher, but since most modern 3D capturing systems have a resolution of at least 640 px × 480 px we did not pursue this issue further.

We distinguished three approaches. Approach A provides an initial estimate for the minimal required spatial resolution and is used as input for approaches B and C. Approach B focuses on the shape of the object of interest, and thus should be used if the shape of the object is the relevant factor. Approach C focuses on a specific area of research, in our case, the symmetry line. The results from approaches B and C range within a similar interval, and thus no further investigation was carried out. We recommend that approaches B and C are always followed. If the results from approaches B and C are not similar, it is up to the researcher to select the criteria for the choice of the minimal required spatial resolution. In the future, we could expand our methods and code to allow the entry of some design parameters and combine approaches A, B, and C into a single evaluation that yields a single result.

This paper presents a practical approach. In particular, the assumption that the Photoneo MotionCam-3D is a valid choice for capturing ground-truth 3D data could be investigated further. Furthermore, the assumption of a five-times oversampling, which is based on practical experience and practical applications found in the literature, requires further research. The influence of the camera system on the signal, and thus the contained frequencies from approach A could be investigated further. This could then be used to achieve a better understanding of the required oversampling.

The choice of practical oversampling, design choice for acceptable errors, sampling artifacts and noise of the camera, surface of the object of interest (e.g., skin color), and distance and viewing angle between camera and object cause uncertainty in the results. Furthermore, our results are mean values (MAE); in the future, we could also quantify the uncertainty, as shown in Figure 11 (left). For the cut-off frequency of 0.02 mm^−1^, the MAE is 6.6 mm; however, the individual error can be as large as 30 mm. In this uncertainty quantification, we could also include models for camera noise, especially for approach A, using a transfer function for the camera, which could improve the result.

Clearly, there are more influential factors to consider in practical applications such as environmental conditions. All measurements were carried out indoors in an illuminated room but with no direct sunlight. In particular, Intel D415 was strongly influenced by ambient light; the darker the room, the smaller the accuracy error.

The approaches and recommendations in this paper can be used to obtain an approximate estimate of the minimal required spatial resolution and the minimal required camera and projector resolution. However, the authors recommend additional practical measurements with such systems to confirm the requirements for individual applications. To support these practical measurements, we have provided the full code.

## 5. Conclusions

The presented methods, code, and results provide researchers with a practical approach to selecting a suitable 3D scanning setup. In our case, the minimal required spatial resolution to capture the human back is between 2 mm and 5 mm, and thus the authors recommend a 3D capturing system with an accuracy error of less than 2 mm. The minimal required camera resolution to capture the human back is 136 px–339 px × 154 px–385 px. Most modern 3D capturing systems have a resolution of at least 640 px × 480 px, and thus, the authors recommend a 3D capturing system with a resolution of at least 640 px × 480 px. However, the minimal required camera resolution is smaller than the concluded resolution.

## Figures and Tables

**Table 1:** Shape quality reduction methods.

Method	MATLAB Method	Description
Downsampling	pcdownsample	Random downsampling
Reduction in spatial frequencies	Butter/filtfilt	Filtering with a low-pass filter
Limiting depth resolution	round	Rounding to the next allowed value
Adding random spatial noise	rand	Adding random spatial noise
Adding sinusoidal spatial noise	Sin	Adding spatial noise in sinusoidal form

**Table 2:** Systems evaluated in this paper.

System	Resolution	Accuracy
Photoneo MotionCam-3D M+	1680 × 1200 resp. 1120 × 800	error < 0.3 mm at 0.9 m
SL/AS with TIDA and HIKROBOT	912 × 1140 resp. 1920 × 1200	error ~1 mm at 1 m
Intel D415	1280 × 720	error < 2% up to 2 m
Orbbec Astra Mini	640 × 480	error < 3 mm at 1 m

**Table 3:** The dependency of minimal required spatial resolution on used oversampling.

Used Oversampling	Corresponding Frequency Factor	Resulting Minimal Required Spatial Resolution
1× (Nyquist limit)	2×	10 mm–25 mm
5×	10×	2 mm–5 mm
10×	20×	1 mm–2.5 mm

## Data Availability

The data presented in this study are openly available in the ETH Research Collection at https://doi.org/10.3929/ethz-b-000615667 (accessed on 5 September 2023).

## Supplementary Materials

The MATLAB code presented in this study is openly available on GitHub at https://github.com/mkaisereth/MinimalRequiredResolution (accessed on 5 September 2023).
